# The complete mitochondrial genome of *Hydrotaea* (*Ophyra*) *chalcogaster* (Diptera: Muscidae)

**DOI:** 10.1080/23802359.2018.1507637

**Published:** 2018-08-28

**Authors:** Jie Zhang, Shixiong Deng

**Affiliations:** Department of Forensic Medicine, College of Basic Medical, Chongqing Medical University, Chongqing, China

**Keywords:** Mitochondrial genome, *Hydrotaea (Ophyra) chalcogaster*, Muscidae

## Abstract

*Hydrotaea* (*Ophyra*) *chalcogaster* (Diptera: Muscidae) is a significant flesh fly in forensic entomology. In this study, the complete mitochondria gene (mitogenome) of *H. chalcogaster* was sequenced and annotated for the first time, and the full-length was a 15,279 bp fragment, consisting of A (39.6%), G (9.1%), T (39,0%), and C (12.3%), which is the classical structure for insect mitogenome. Phylogenetic analyses showed that *H. chalcogaster* clearly separated from the Muscinae subfamily. This work provides support for further study of the use of mitochondrial genome in the species identification, and enriches the databases of the Muscidae species.

*Hydrotaea chalcogaster* (Wiedemann, 1824) is a significant flesh fly for forensic investigations (Carvalho et al. 2000). Their utility was severely limited as the highly similar morphological appearance. With the development of molecular identification, the mitochondrial genome (mitogeome) has been widely used for species identification (Byrd and Castner 2009).

Adult specimens of *H. chalcogaster* were captured in Changsha, Hunan province, China, in July 2017. The *H. chalcogaster* mitogenome has been submitted to GenBank with accession number is MH521131.

The total mitogenome was extracted using the CTAB (Cetyl Trimethyl Ammonium Bromide) method (Skevington and Yeates 2000). The complete mitogenome of *H. chalcogaster* was amplified with the overlapping short PCR primers. PCR amplicons were carried out following the cyclic conditions with TaKaRa LA Taq® (TaKaRa, Dalian, China). PCR products were sequenced directly by an ABI PRISM 3730 automated sequencer (Applied Biosystems, Foster, USA). The sequence files were manually proofread and assembled into contigs with BioEdit 7.0.9.0 (Hall 1999). In order to confirm the correctness of gene boundaries, all genes of full-mitogenome were respectively aligned with other muscid as implemented in MEGA 7.0 (Kumar et al. 2016).

Phylogenetic analyses of 10 Muscidae species were performed based on the complete mitogenome sequences using Neighbor-joining inference methods, with two Sarcophagidae species as outgroup (Figure 1). Phylogenetic analyses showed that *H. chalcogaster* clearly separated from the muscinae subfamily.

**Figure 1.**
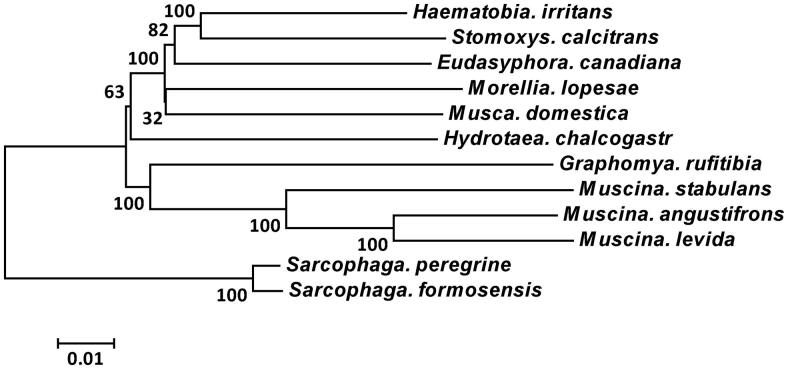
Phylogenetic analyses were constructed using Neighbor-joining inference methods based on complete mitogenome sequences, with two Sarcophagidae species as outgroup. Numbers at nodes are bootstrap values. Evolutionary distance divergence scale bar is 0.01.
